# Kinesio Taping for Balance Function after Stroke: A Systematic Review and Meta-Analysis

**DOI:** 10.1155/2019/8470235

**Published:** 2019-07-16

**Authors:** Yijuan Hu, Dongling Zhong, Qiwei Xiao, Qiang Chen, Juan Li, Rongjiang Jin

**Affiliations:** ^1^Chengdu University of Traditional Chinese Medicine, Chengdu, Sichuan Province, China; ^2^Huili County People's Hospital, Sichuan Province, China

## Abstract

**Objective:**

With the increasing social and economic burdens of balance impairment after stroke, the treatment for balance impairment after stroke becomes a major public health problem worldwide. Kinesio taping (KT) as a part of clinical practice has been used widely in the treatment of balance impairment after stroke. However, the clinical effects of KT for balance function have not been confirmed. The objective of this study is to investigate the effects and safety of KT for balance impairment after stroke.

**Methods:**

We conducted a systematic review (SR) and meta-analysis of randomized controlled trials (RCTs) on the effects of KT for balance impairment after stroke. We searched the following databases: (1) English databases: EMBASE (via Ovid), MEDLINE (via Ovid), the Cochrane library, PubMed, and PEDro; (2) Chinese databases: China Biology Medicine (CBM), Wan Fang database, China National Knowledge Infrastructure (CNKI), and VIP. Besides, hand searches of relevant references were also conducted. We systematically searched from the inception to December 2018, using the keywords (Kinesio, Kinesio Tape, tape, or Orthotic Tape) and (stroke, hemiplegia, or hemiplegic paralysis) and (balance or stability). The search strategies were adjusted for each database. The reference lists of included articles were reviewed for relevant trials. For missing data, we contacted the authors to get additional information.

**Results:**

22 RCTs involved 1331 patients, among which 667 patients in the experimental group and 664 patients in the control group were included. Results of meta-analysis showed that, compared with conventional rehabilitation (CR), there was significant difference in Berg Balance Scale (BBS) (MD=4.46, 95%CI 1.72 to 7.19,* P*=0.001), Time Up and Go Test (TUGT) (MD=-4.62, 95%CI -5.48 to -3.79,* P *< 0.00001), functional ambulation category scale (FAC) (MD=0.53, 95%CI 0.38 to 0.68,* P *< 0.00001), Fugl-Meyer assessment (FMA-L) (MD=4.20, 95%CI 3.17 to 5.24,* P *< 0.00001), and Modified Ashworth Scale (MAS) (MD=-0.38, 95%CI -0.49 to -0.27,* P *< 0.00001). The results of subgroup analysis showed that there was no significant difference between KT and CR with ≤4 weeks treatment duration (< 4 weeks: MD=5.03, 95%CI -1.80 to 11.85,* P*=0.15; =4 weeks: MD=4.33, 95%CI -1.50 to 10.15,* P*=0.15), while there was significant difference with more than 4-week treatment duration (MD=4.77, 95%CI 2.58 to 6.97,* P *< 0.0001).

**Conclusions:**

Based on current evidence, KT was more effective than CR for balance function, lower limb function, and walking function in poststroke patients. Longer treatment duration may be associated with better effects. However, more well-conducted RCTs are required in the future.

## 1. Introduction

Stroke is a common clinical cerebrovascular disease, including cerebral infarction, intracerebral hemorrhage (ICH), and subarachnoid hemorrhage (SAH), with a high morbidity, which is a major cause of disability and death among people around the world [1]. As the world's population ages, the burden of stroke increases rapidly [2]. WHO Global status report on noncommunicable disease (NCD) showed an estimated 6.7 million NCD deaths were caused by stroke; 33 million of stroke survivors needed long-term follow-up and secondary preventive measures [3]. A study on 84,184 acute stroke patients in England, Wales, and Northern Ireland showed that the total cost of health and social care each year was £3.60 billion in the first five years after admission [4]. A study on economic burden of informal care attributable to stroke among those aged 65 years or older in China showed that the average annual cost of informal care associated with stroke was 10,612 RMB per stroke survivor [5].

Balance impairment is one of the common daily functional problems in stroke patients, which seriously affects the patient's daily life and work. According to the severity of stroke, the manifestations of balance impairment can be classified into stepping, standing, and sitting balance impairment [6]. Poor balance ability is often associated with increased risk of falls, disability, and even mortality. A study based on 41 community-dwelling people with stroke showed 50% (21/41) participants were classed as fallers, of whom 10 had fallen repeatedly [7]. Another study based on 522 adults showed that, by 2 years after stroke, 23.5% (124/522) adults had fallen at least once, 14.2% (74/522) had 2 or more falls and 5.4% (28/522) had a fracture [8]. Especially for old people, owing to poor balance ability, they have to face the risk of body injuries caused by falls for a long time [9], some severe falls can even lead to paralysis or death. This damage is undoubtedly serious and complicated. Hence, it is urgent for stroke patients to improve their balance ability [10–15].

Kinesio taping (KT) was introduced by Kenzo Kase in 1996, which normally involves a combination of applying appropriate tension along the elastic therapeutic tape and placing the target muscle in a stretched position and is widely used as an interesting and relatively novel method for various clinical treatments. KT has gained popularity in clinical practice and been used in clinical practice worldwide. By supporting weak muscle, relaxing overstretched muscle, and reducing pain, KT can promote functional use of the upper and lower extremity, in further to improve balance ability [16]. Besides, KT was also a good option for stroke patients who had asymmetrical and imbalanced body posture [17]. Recent studies showed that KT has been used as an adjuvant rehabilitation therapy in the treatment of balance impairment after stroke [18–36], but the conclusions were inconsistent. The effect of KT on balance impairment of poststroke patients is still controversial. Therefore, a systematic review (SR) and meta-analysis of randomized controlled trials (RCTs) was performed to investigate the efficacy and safety of KT on balance impairment after stroke.

## 2. Materials and Methods

### 2.1. Data Sources and Searches

We searched the following databases:

(1) English databases: EMBASE (via Ovid), MEDLINE (via Ovid), the Cochrane library, PubMed, and PEDro;

(2) Chinese databases: China Biology Medicine (CBM), Wan Fang database, China National Knowledge Infrastructure (CNKI), and VIP.

Hand searches of relevant references were conducted. Clinical trials registration websites, including https://clinicaltrials.gov/ and http://www.chictr.org.cn, were also searched for possible RCTs.

All the databases were conducted from their inception to December 2018. The search terms (Kinesio, Kinesio Tape, tape, or Orthotic Tape) and (stroke, hemiplegia, or hemiplegic paralysis) and (balance or stability) were used and the search strategies were adjusted for each database. Details of search strategies were given in Appendix A.

### 2.2. Inclusion Criteria

Trials were screened based on the following inclusion criteria: (1) RCTs of KT for stroke patients with balance impairment; (2) patients with balance impairment after stroke diagnosed according to stroke diagnostic criteria with clear history and manifestations and confirmed by the brain Computed Tomography (CT) or Magnetic Resonance Imaging (MRI) and the age and sex are not limited; (3) the experimental group was treated with KT along with or without conventional rehabilitation (CR). The control group can be CR, sham KT, or placebo; (4) primary outcome is Berg Balance Scale (BBS). Secondary outcomes include related outcomes to lower limb function, spasticity, walking function.

### 2.3. Exclusion Criteria

Trials met the following criteria would be excluded: (1) Trials were non-RCTs, such as literature review, case report, and expert treatment experience summary; (2) balance impairment was not caused by stroke, for example, caused by Parkinson's disease, pediatric cerebral palsy, knee surgery, or other diseases; (3) duplicate or the data cannot be extracted.

### 2.4. Studies Selection

All the retrieved studies were imported into Endnote (X8), and the filter tool was used to delete the duplicated studies. Two reviewers (YJH and DLZ) independently screened the titles and abstracts according to the inclusion and exclusion criteria. After screening, two reviewers (YJH and DLZ) cross checked and downloaded the full text of all possibly relevant studies for further assessment. The disagreements were resolved through team discussion.

### 2.5. Data Extraction

A standardized data extraction form was designed in advance. The following information of the included studies, first author; publication year; country of origin; participant characteristics; evaluation time and outcomes, were independently extracted by two reviewers (QWX and YJH). If the trials had more than 2 groups or factorial designs and multiple comparisons, we extracted only the information and data of interest reported in the original articles. In case of disagreements, a third reviewer (RJJ) was involved.

### 2.6. Risk-of-Bias Assessments

The methodological quality for the included RCTs was independently assessed by 2 reviewers (YJH and QC) based on Cochrane risk-of-bias criteria [19]. The Cochrane risk-of-bias criteria included the randomization sequence generation, allocation concealment, blinding of participants and personnel, blinding of outcome assessment, incomplete outcome data, selective reporting, and other bias. Each quality item was graded as low, high, or unclear. The disagreements of assessment were resolved through team discussion.

### 2.7. Statistical Analysis

We performed meta-analysis to calculate mean difference (MD) and 95% CIs using the Mantel-Haenszel statistical method. Heterogeneity test of each outcome was conducted by using the Chi-square test with no significance (*P*>0.05), and *I*^2^ statistic evaluated the degree of heterogeneity. If *I*^2^ < 50%, many similar studies could be considered to have no homogeneity, the fixed effect model adopted for a meta-analysis; otherwise random effects model was performed. If substantial heterogeneity was detected, subgroup analysis or sensitivity analysis could be applied to explore the causes of heterogeneity. If the sources of heterogeneity could not be determined, descriptive analysis was adopted.

## 3. Results

### 3.1. Study Selection

**Figure 1:**
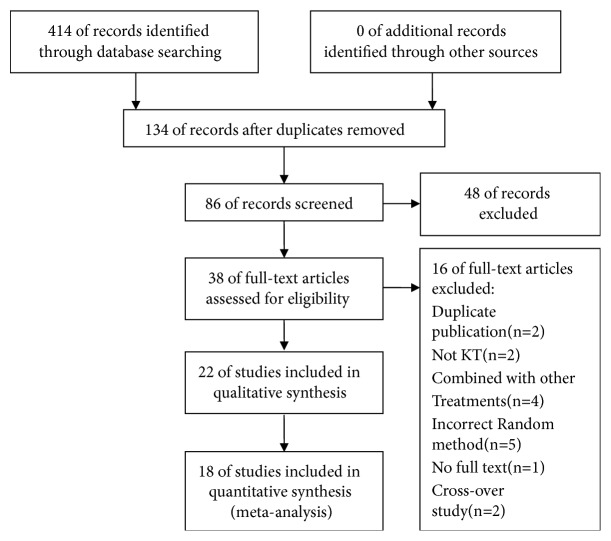
PRISMA flow chart for study selection.

A total of 414 potentially relevant articles were retrieved. After removing duplicates, titles and abstracts of remaining articles were screened for inclusion. After reading full texts, 22 RCTs were included in SR and 18 RCTs for meta-analysis. Excluded articles with explanations were listed in Appendix B. Flow diagram for selection of the included studies was showed in Figure 1.

### 3.2. Studies Description

22 RCTs involved 1331 patients, among which 667 patients are in the experimental group and 664 patients are in the control group. Included RCTs were published from 2014 to 2019, among which 18 RCTs were conducted in China, 3 were in Korean [18–20], and 1 was in Iran [21]. Disease duration varied from days to years. The control group in 20 RCTs utilized CR, while 2 RCTs used sham KT [20, 22]. The locations of KT were on lower limbs (quadriceps, triceps surae, tibialis anterior, ankle, and so on). The treatment duration of KT varied from immediate effect to 3 months. Only 1 RCT mentioned follow-up [22]; only 7 RCTs reported funding and sources [21, 25, 28, 32, 35–37]. The characteristics of the included RCTs in detail were showed in Table 1.

### 3.3. Methodological Quality

**Figure 2:**
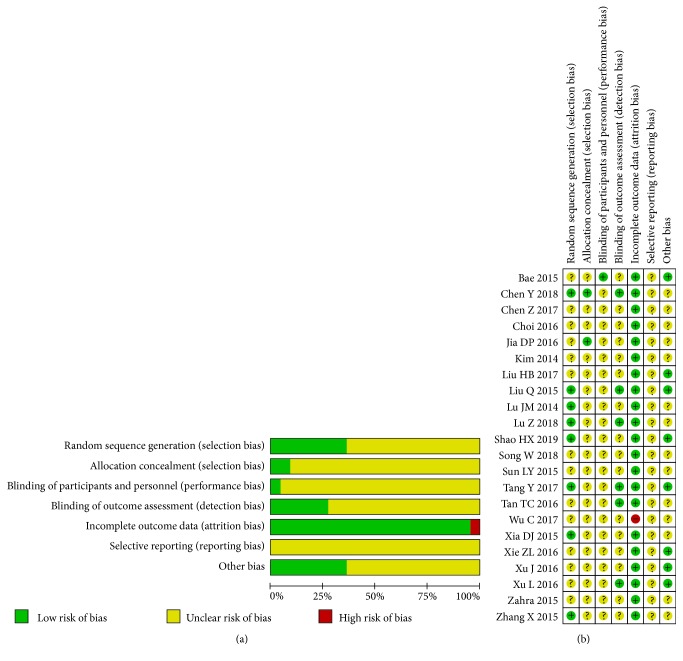
(a, b) Risk of bias of included studies.

The risk of bias assessed by the Cochrane Collaboration's tool of the included RCTs was summarized in Figures 2(a) and 2(b). Among the 22 included RCTs, the randomization procedure was reported adequately in the 8 RCTs; 2 RCTs clearly reported the allocation concealment. Only 1 RCT described the blinding of participants and personnel. We considered RCTs using objective outcome indicator as “low quality”; there were 6 RCTs considered “low quality” in the blinding of outcome assessment. In incomplete outcome data section, only 1 RCT was considered “high quality” due to no reason for loss. All the 22 RCTs were rated “uncertain risks” in selective reporting for no protocol published in advance. In other bias section, we considered “uncertain risks” if the study did not report conflict of interest or source of funds.

### 3.4. Meta-Analysis

#### 3.4.1. Primary Outcome

**Figure 3:**
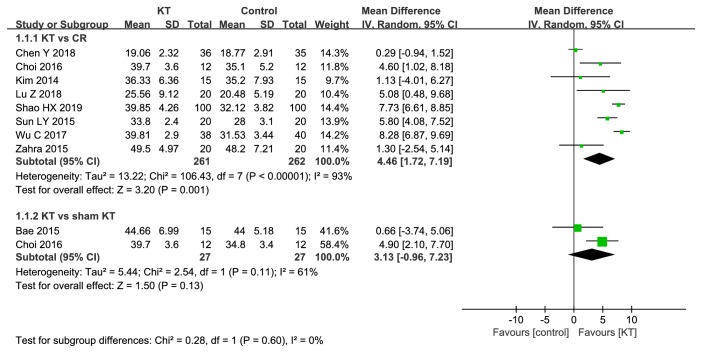
Meta-analysis results of KT for BBS.

*BBS. *8 RCTs compared KT with CR [18, 19, 21, 22, 26, 28, 30, 33]. As shown in Figure 3, there was significant difference between KT and CR in BBS (MD=4.46, 95%CI 1.72 to 7.19,* P*=0.001); 2 RCTs compared KT with sham KT, there was no significant difference (MD=3.13, 95%CI -0.96 to 7.23,* P*=0.13). We performed sensitivity analysis by removing RCTs one by one; the results remained unchanged.

**Figure 4:**
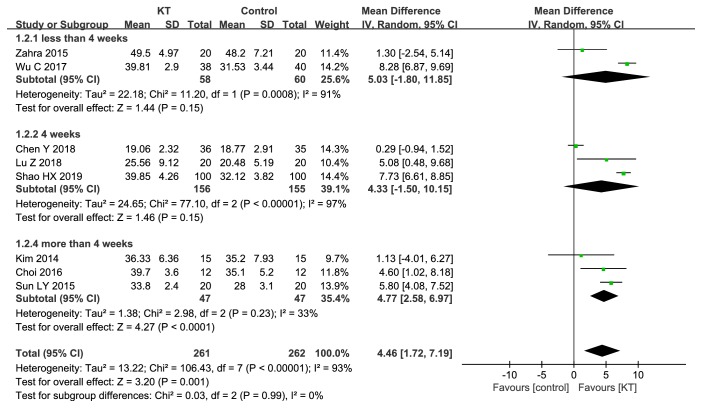
Subgroup analysis of KT for BBS.

Subgroup analysis was performed based on the treatment duration. The results showed that there was no significant difference between KT and CR with treatment duration ≤4 weeks (MD=5.03, 95%CI -1.80 to 11.85,* P*=0.15; MD=4.33, 95%CI -1.50 to 10.15,* P*=0.15), while there was significant difference with treatment duration >4weeks (MD=4.77, 95%CI 2.58 to 6.97,* P*<0.0001). Subgroup analysis was showed in Figure 4.

#### 3.4.2. Secondary Outcomes

**Figure 5:**
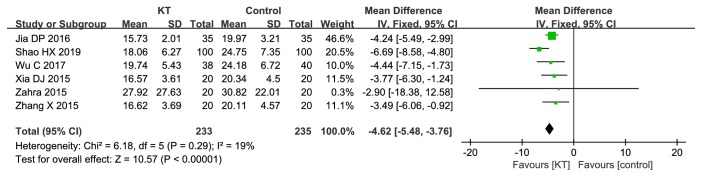
Meta-analysis results of KT for TUGT.

*TUGT. *6 RCTs compared KT with CR [21, 23, 28, 33, 34, 38]. As shown in Figure 5, there was significant difference in TUGT between KT and CR (MD=-4.62, 95%CI -5.48 to -3.76,* P*<0.00001). We performed sensitivity analysis by removing RCTs one by one, the results remained unchanged.

**Figure 6:**
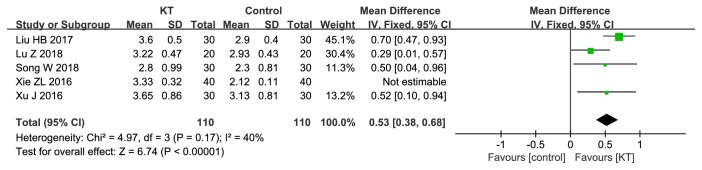
Meta-analysis results of KT for FAC.

*FAC. *5 RCTs compared KT with CR [24, 26, 29, 35, 36]. As shown in Figure 6, there was significant difference in FAC between KT and CR (MD=0.53, 95%CI 0.38 to 0.68,* P*<0.00001). We performed sensitivity analysis by removing RCTs one by one, and the results remained unchanged. However, by removing Xie's study, the* I*^2^ decreased from 93% to 40%.

**Figure 7:**
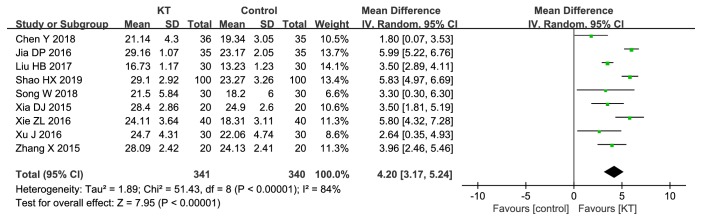
Meta-analysis results of KT for FMA-L.

*FMA-L. *9 RCTs compared KT with CR [22–24, 28, 29, 34–36]. As shown in Figure 7, there was significant difference in FMA-L between KT and CR (MD=4.20, 95%CI 3.17 to 5.24,* P*<0.00001). We performed sensitivity analysis by removing RCTs one by one, and the results remained unchanged.

**Figure 8:**
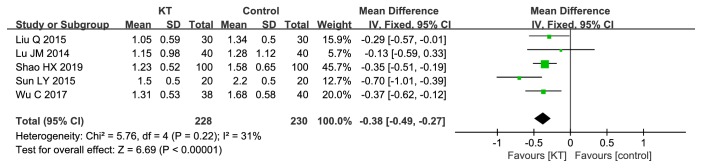
Meta-analysis results of KT for MAS.

*MAS. *5 RCTs compared KT with CR [25, 27, 28, 30, 33]. As shown in Figure 8, there was significant difference between KT and CR in MAS (MD=-0.38, 95%CI -0.49 to -0.27,* P*<0.00001). We performed sensitivity analysis by removing RCTs one by one, and the results remained unchanged.

*Other Outcomes. *As shown in Table 2, there was significant difference between KT and CR in 10mMVS (MD=0.23, 95%CI 0.06 to 0.39,* P*=0.006), MMT (MD=0.35, 95%CI 0.10 to 0.59,* P*=0.005), AROM (MD=1.98, 95%CI 1.16 to 2.80,* P *< 0.00001) and Brunnstrom (MD=0.32, 95%CI 0.07 to 0.57,* P*=0.01). However, there was no significant difference between KT and CR in 10mMWT (MD=-3.68, 95%CI -15.97 to 8.61,* P*=0.56). Xu L et al. [37] found that KT was more effective than CR on lower limb swelling in stroke patients. Chen Z et al. [39] reported that KT can effectively decrease the angle of strephenopodia; it was safe with no adverse effects. Besides, Tan TC et al. [31] found that KT had a positive effect in improving gait and walking ability of hemiplegia patients. Tang Y et al. [32] found KT with physical therapy may be favorable in improving functional outcome of sit-to-stand transfer in stroke patients and use on both sides is more effective.

## 4. Discussion

### 4.1. Summary of Findings

To our knowledge, this is the first meta-analysis of KT for balance ability after stroke. Results of this meta-analysis showed that KT was more effective than CR for balance ability, lower limb function, and walking function in poststroke patients. But no significant difference was found between KT and sham KT in BBS. Possible causes may be only two studies included. However, the results of BBS were not consistent regarding of the treatment duration. In the subgroup analysis, we found that, compared with CR, KT was more effective with more than 4-week treatment duration. However, there was no significant difference between KT and CR with treatment duration ≤4 weeks, which suggested that long-term KT may be more effective than short-term KT. Except 10mMWT, there was significant difference between KT and CR in TUGT, FMA, FAC, MAS, and other outcomes.

### 4.2. Implications for Further Studies

Balance impairment is one of the common daily functional problems in stroke patients, which is often associated with increasing risk of falls, poor walking function and low quality of life. KT is a safe and effective method, while the mechanism of KT is not clear at present, its therapeutic effect may be the stickiness and principles of kinematics and biomechanics, by sticking KT into different directions and using different tensions. Recent studies showed that KT had been used as an adjuvant rehabilitation therapy in the treatment of balance impairment after stroke. We retrieved 4 SRs of the clinical effects of KT [40–43], 1 SR was not only focus on stroke patients [40], and 3 of them focused on the motor function of lower extremity, which directly affected the balance function [41–43]. Compared with other outcomes, balance ability is the key for poststroke patients to return to family and society. 1 previous SR concluded that the current existing evidence was insufficient to support the use of KT over other modalities in clinical practice [40]. Due to these controversial conclusions, our SR focused on balance function and made a more comprehensive search in English and Chinese databases. 22 RCTs which met the inclusion criteria were included.

Results of Wu YH et al. [43] showed that 4-week KT was more effective than 6 weeks KT, which was opposite to our results. The possible reasons for the opposite result may be that Wu YH et al. combined BBS and TUGT together, which certainly contributed to heterogeneity. Our SR took BBS as primary outcome and TUGT as secondary outcome. Results of our SR indicated that KT was superior to CR in both BBS and TUGT.

### 4.3. Strengths and Limitations

This SR provides the latest evidence on KT for balance function after stroke based on the findings of relevant RCTs; the results of our meta-analysis showed that KT may be a beneficial complementary therapy in the balance rehabilitation process for stroke patients. The results of this study will provide evidence for the clinical application of KT. Different from current SRs, we conducted a comprehensive search and reporting in accordance with PRISMA (Appendix C). However, our study still has several limitations. First of all, the treatment duration of KT was inconsistent, from as short as an immediate treatment to 12 weeks, indicating a wide variation in designs. Second, the locations of KT were not standardized in lower limb, hip, thigh, and crus, which may also lead to discrepancy. Third, several outcomes included only 2 studies, which may lead to unreliable results.

Hence, more high methodological quality, larger sample size, and standard-designed trials are required to draw a definitive conclusion and provide a standard KT program.

## 5. Conclusion

Based on current evidence, this study demonstrated that KT was more effective than CR for balance ability, lower limb function and walking function in poststroke patients. Longer treatment duration may be associated with better effects. However, more well-conducted RCTs are required in the future.

## Figures and Tables

**Table 1:** Characteristics of included studies.

No.	First Author	Participants			Intervention			Outcome	Fund
	Year								
	Country								
		Age	Sex	Disease Duration	Intervention	KT POS	Treatment Duration		
			(M/F)						
1	Wu C 2017 China	T:56.20±8.31 C:54.60±9.16	T:22/16 C:25/15	T:5.98±2.28d C:6.35±2.43d	T:CR+KT C:CR	Quadriceps Triceps surae Tibialis anterior	5d	①②⑦⑧	-
2	Sun LY 2015 China	53.2±17.1	22/18	-	T:CR+KT C:CR	Triceps surae Tibialis anterior Ankle	6w	①⑦⑨	-
3	Shao HX 2019 China	T:53.28±4.59 C:54.38±5.06	T:55/45 C:52/48	T:5.46±1.25d C:5.17±1.05d	T:CR+KT C:CR	Quadriceps Triceps surae Tibialis anterior	4w	①②③⑦⑧	Yes
4	Lu Z 2018 China	T1:62.89±8.71 C:61.25±5.18	T1:13/7 C:12/8	T1:3.12±1.87w C:4.15±1.53w	T1:CR+KT C:CR+walk training	Triceps surae Tibialis anterior peroneus longus Peroneus brvis	4w	①⑤⑥⑨	-
5	Chen Y 2018 China	T:55.42±13.55 C:56.17±13.50	T: 18/18 C: 18/17	T:14.72±1.16 C:14.40±1.14	T:CR+KT C:CR+sham KT	Triceps surae Tibialis anterior Pain and swelling point	4w	①③⑥	-
6	Zahra 2015 Iran	T:49.3±9.3 C:49.3±10.6	T: 14/6 C: 11/9	T:1.4 ±0.8y C:1.2 ±0.5y	T:KT C:-	Hip Knee Ankle	24h	①②	Yes
7	Jia DP 2016 China	T:50.92±4.71 C:51.93±4.71	T: 18/17 C: 19/16	T:1.72 ±0.35m C:1.96 ±0.27m	T:CR+KT C:CR	Tibialis anterior Ankle	4w	②③⑤	-
8	Liu HB 2017 China	T:56.73±8.51 C:57.77±6.74	T: 17/13 C: 16/14	T:30.53 ±4.60d C:32.43 ±4.80d	T:CR+KT C:CR	Quadriceps Hamstring Triceps surae Tibialis anterior	2m	③⑥	-
9	Liu Q 2015 China	T:68.8±6.8 C:67.9±5.4	T: 18/12 C: 20/10	T:18.8 ±4.07d C:20.43 ±5d	T:CR+KT C:CR	Calf Ankle Foot	1m	③④⑦	Yes
10	Lu JM 2014 China	T:59.13±11.60 C:59.18±11.46	T: 19/21 C: 17/23	T:2.6m C:2.7m	T:CR+KT C:CR	Calf Ankle Foot	5d	⑦⑩	-
11	Song W 2018 China	T:60.03±8.56 C:61.63±9.56	T: 16/14 C: 18/12	T:39.20±17.14d C:37.90±18.40d	T:CR+KT C:CR	Calf Ankle Foot	4w	③⑥⑩	-
12	Xia DJ 2015 China	T:52.2±3.94 C:52.2±4.49	T: 15/5 C: 14/6	T:1.82±1.29m C:2.20±0.71m	T:CR+KT C:CR	Calf Ankle Foot	4w	②③⑤	-
13	Xie ZL 2016 China	T:55.32±13.21 C:56.36±12.11	T: 24/16 C: 18/22	T:31.55±12.05d C:33.18±10.11d	T:CR+KT C:CR	Thigh Calf Ankle Foot	12w	③⑥	Yes
14	Xu J 2016 China	T:64.40±10.23 C:66.26±10.00	T: 18/12 C: 16/14	T:87.70±36.31d C:92.83±40.21d	T:CR+KT C:CR	Calf Ankle Foot	8w	③⑥	Yes
15	Zhang X 2015 China	T:33~80 C:35~78	T: 11/9 C: 10/10	≤12m	T:CR+KT C:CR	Calf Ankle Foot	4w	②③	-
16	Bae 2015 Korea	T:65.08±9.33 C:63.50±5.90	T: 15 C: 15	-	T:KT C:placebo taping	Fibularis longus Fibularis tertius Extensor digitorum Longus Tibialis anterior	Immediately	①	-
17	Choi 2016 Korea	-	T1:12 C: 12	≥6m	T1:PNF+KT C:PNF	rectus femoris muscle and the tensor fasciae latae	8w	①④	-
18	Kim 2014 Korea	-	T: 15 C: 15	-	T:CR+KT C:CR	quadriceps femoris tibialis anterior	6w	①④	-
19	Tan TC 2016 China	T:60.3±7.9 C:58.3±8.9	T: 7/12 C: 9/10	T:56.9±16.5d C:58.2±14.6d	T:CR+KT C:CR	Calf Ankle Foot	1m	Gait parameters	--
20	Tang Y 2017 China	T:52.8±3.86 C:52.6±4.03	T: 14/6 C: 13/7	T:1.75±0.78m C:1.82±0.91m	T:CR+KT C:CR	Hip	6w	foot pressure device	Yes
21	Xu L 2016 China	T:62.56±0.04 C:62.32±0.13	T: 23/22 C: 24/21	-	T:CR+KT C:CR	Calf Ankle Foot	20d	Swelling	Yes
22	Chen Z 2017 China	T:45~72 C:46~70	T: 12/18 C: 14/16	T:6~15m C: 6~15m	T:CR+KT C:CR	Calf Ankle Foot	2m	Angle of strephenopodia CSI	-

Note: T: experimental group; C: control group; POS: position;

① BBS: Berg Balance Scale; ② TUGT: Timed Get Up and Go Test; ③ FMA-L: Fugl-Meyer motor assessment of lower limb; ④ 10mMWT: 10 m walking test; ⑤ 10mMWS: 10m walking speed; ⑥ FAC: functional ambulation category; ⑦ MAS: Modified Ashworth Scale; ⑧ MMT: manual muscle test; ⑨ AROM: active range of motion; ⑩ Brunnstrom.

**Table 2:** Meta-analysis results of KT for other outcomes.

Outcome	No. of RCT	MD	95%CI	*P* value
10mMVS	3	0.23	0.06, 0.39	0.006
10mMWT	3	-3.68	-15.97, 8.61	0.56
MMT	2	0.35	0.10, 0.59	0.005
AROM	2	1.98	1.16, 2.80	<0.00001
Brunnstrom	2	0.32	0.07, 0.57	0.01

**Table 3:** 

No.	First Author	Title	Reason
1	Mo XX	Clinical efficacy evaluation of electroacupuncture combined with early intervention of kinesio taping for foot drop in patients with ischemic stroke	KT+ electroacupuncture

2	Li YB	Effect of kinesio taping combined with local injection of botulinum toxin type A and routine rehabilitation training on walking ability of patients with lower limb paralysis after stroke	KT+ local injection of botulinum toxin type A

3	Li WL	Therapeutic effect of Frenkel gymnastics training combined with kinesio taping on patients with post-stroke ataxia	KT+ Frenkel gymnastics training

4	Huang YT	Clinical Observation on the Therapeutic Effect of Acupuncture and Moxibustion Combined with Intramuscular Patching for Treatment of Post-stroke Foot Inversion	KT+ acupuncture

5	He LW	Clinical observation of abnormal gait in stroke using kinesio taping	Non-RCT

6	Chen B	The effects of kinesio taping on strephenopodia after stroke patients	Non-RCT

7	Timur Ekiz	Effects of Kinesio Tape application to quadriceps muscles on isokinetic muscle strength, gait, and functional parameters in patients with stroke	Non-RCT

8	Belma Fusun Koseoglu	Can kinesio tape be used as an ankle training method in the rehabilitation of the stroke patients?	Non-RCT

9	Xiao Y	Effect of intramuscular effect on recovery of foot drop in stroke	Non-RCT

10	Sung Rae Yang	Immediate effects of kinesio taping on fixed postural alignment and foot balance in stroke patients	Cross-over study

11	Gokhan Yazici	Does correcting position and increasing sensorial input of the foot and ankle with Kinesio Taping improve balance in stroke patients?	Cross-over study

12	He JS	Effect of kinesio taping on balance function and lower limbs exercise ability in stroke patients	No full text

13	Stefano Carda	Casting, taping or stretching after botulinum toxin type A for spastic equinus foot: a single-blind randomized trial on adult stroke patients	No KT

14	Clare Maguire	Hip abductor control in walking following stroke−−the immediate effect of canes, taping and TheraTogs on gait	No KT

15	Liu Q	Clinical effects of kinesio taping for treating stroke patients with knee instability and degeneration	Duplicate publication

16	Chen Y	Efficacy of kinesio taping combined with functional training on stroke patients	Duplicate publication

**Table 4:** 

Section/topic	#	Checklist item	Reported on page #
*TITLE *			
Title	1	Identify the report as a systematic review, meta-analysis, or both.	1

*ABSTRACT *			
Structured summary	2	Provide a structured summary including, as applicable: background; objectives; data sources; study eligibility criteria, participants, and interventions; study appraisal and synthesis methods; results; limitations; conclusions and implications of key findings; systematic review registration number.	1-2

*INTRODUCTION *			
Rationale	3	Describe the rationale for the review in the context of what is already known.	3-4
Objectives	4	Provide an explicit statement of questions being addressed with reference to participants, interventions, comparisons, outcomes, and study design (PICOS).	4

*METHODS *			
Protocol and registration	5	Indicate if a review protocol exists, if and where it can be accessed (e.g., Web address), and, if available, provide registration information including registration number.	/
Eligibility criteria	6	Specify study characteristics (e.g., PICOS, length of follow-up) and report characteristics (e.g., years considered, language, publication status) used as criteria for eligibility, giving rationale.	5
Information sources	7	Describe all information sources (e.g., databases with dates of coverage, contact with study authors to identify additional studies) in the search and date last searched.	4-5
Search	8	Present full electronic search strategy for at least one database, including any limits used, such that it could be repeated.	4
Study selection	9	State the process for selecting studies (i.e., screening, eligibility, included in systematic review, and, if applicable, included in the meta-analysis).	5
Data collection process	10	Describe method of data extraction from reports (e.g., piloted forms, independently, in duplicate) and any processes for obtaining and confirming data from investigators.	6
Data items	11	List and define all variables for which data were sought (e.g., PICOS, funding sources) and any assumptions and simplifications made.	6
Risk of bias in individual studies	12	Describe methods used for assessing risk of bias of individual studies (including specification of whether this was done at the study or outcome level), and how this information is to be used in any data synthesis.	6
Summary measures	13	State the principal summary measures (e.g., risk ratio, difference in means).	6
Synthesis of results	14	Describe the methods of handling data and combining results of studies, if done, including measures of consistency (e.g., I^2^) for each meta-analysis.	6

## Appendix

### A. Search Strategy

(1) Tape, Athletic OR Orthotic Tape OR Kinesio Tape OR Kinesio tape

(2) Strokes OR Cerebrovascular Accident OR CVA (Cerebrovascular Accident) OR Cerebrovascular Apoplexy OR Brain Vascular Accident OR Cerebrovascular Stroke OR Apoplexy OR Cerebral Stroke OR Acute Stroke OR Acute Cerebrovascular Accident

(3) (1) AND (2)

### B. Excluded Studies

See Table 3.

### C. PRISMA Checklist

See Table 4.

## References

[1] Sacco L R., Kasner E S., Broderick P J. (2064). An Updated Definition of Stroke for the 21st Century. *Journal of Neurology and Neurorehabilitation*.

[2] Asia Pacific Consensus Forum on Stroke Management Organizing Committees.

[3] Mendis S., Davis S., Norrving B. (2015). Organizational update: the World Health Organization global status report on noncommunicable diseases 2014; one more landmark step in the combat against stroke and vascular disease. *Stroke*.

[4] Xu X., Vestesson E., Paley L. (2017). The economic burden of stroke care in England, Wales and Northern Ireland: Using a national stroke register to estimate and report patient-level health economic outcomes in stroke. *European Stroke Journal*.

[5] Joo H., Liang D. (2017). Economic burden of informal care attributable to stroke among those aged 65 years or older in China. *International Journal of Stroke*.

[6] Tyson S. F., Hanley M., Chillala J., Selley A., Tallis R. C. (2006). Balance Disability After Stroke. *Physical Therapy in Sport*.

[7] Hyndman D., Ashburn A., Stack E. (2002). Fall events among people with stroke living in the community: circumstances of falls and characteristics of fallers. *Archives of Physical Medicine and Rehabilitation*.

[8] Callaly E. L., Ni Chroinin D., Hannon N. (2015). Falls and fractures 2 years after acute stroke: the North Dublin Population Stroke Study. *Age and Ageing*.

[9] Saari P., Heikkinen E., Sakari-Rantala R., Rantanen T. (2007). Fall-related injuries among initially 75- and 80-year old people during a 10-year follow-up. *Archives of Gerontology and Geriatrics*.

[10] Gryfe C. I., Amies A., Ashley M. J. (1977). A longitudinal study of falls in an elderly population: I. Incidence and morbidity. *Age and Ageing*.

[11] Wild D., Nayak U. S., Isaacs B. (1981). Prognosis of falls in old people living at home. *Journal of Epidemiology & Community Health*.

[12] Baker S. P., Harvey A. H. (1985). Fall Injuries in the Elderly. *Clinics in Geriatric Medicine*.

[13] Tinetti M. E. (1986). Performance-orientated assessment of mobility problems in elderly patients. *Journal of the American Geriatrics Society*.

[14] Tinetti M. E., Speechley M., Ginter S. F. (1988). Risk factors for falls among elderly persons living in the community. *The New England Journal of Medicine*.

[15] Nyberg L., Gustafson Y. (1995). Patient falls in stroke rehabilitation: A challenge to rehabilitation strategies. *Stroke*.

[16] Jaraczewska E., Long C. (2006). Kinesio taping in stroke: Improving functional use of the upper extremity in hemiplegia. *Topics in Stroke Rehabilitation*.

[17] Yang S. R., Heo S. Y., Lee H. J. (2015). Immediate effects of kinesio taping on fixed postural alignment and foot balance in stroke patients. *Journal of Physical Therapy Science*.

[18] Kim W. I., Choi Y. K., Lee J. H., Park Y. H. (2014). The effect of muscle facilitation using kinesio taping on walking and balance of stroke patients. *Journal of Physical Therapy Science*.

[19] Choi Y.-K., Park Y.-H., Lee J.-H. (2016). Effects of kinesio taping and McConnell taping on balance and walking speed of hemiplegia patients. *Journal of Physical Therapy Science*.

[20] Bae Y.-H., Kim H. G., Min K. S., Lee S. M. (2015). Effects of Lower-Leg Kinesiology Taping on Balance Ability in Stroke Patients with Foot Drop. *Evidence-Based Complementary and Alternative Medicine*.

[21] Rojhani-Shirazi Z., Amirian S., Meftahi N. (2015). Effects of ankle kinesio taping on postural control in stroke patients. *Journal of Stroke and Cerebrovascular Diseases*.

[22] Chen Y. (2018). *Effects of kinesio taping on lower limb function in patients with hemiplegia after early stroke*.

[23] Jia D. P., Li L., Wu Z. Q. (2016). Effect of intramuscular effect on the walking function of stroke patients with hemiplegia. *China Medical Equipment*.

[24] Liu HB., Cai P., Xiong Y. (2017). Efficacy of kinesio taping in the improvement on walking ability of stroke patients with knee hyperextension. *Chinese Journal of Trauma and Disability Medicine*.

[25] Liu Q., Tian Q., Yu B. (2015). Clinical observation of kinesio taping with physical therapy for improving functional outcome of stroke patients. *Geriatrics & Health Care*.

[26] Lu Z., Tang Y., Hu FD. (2018). Effect of triggered functional electrical stimulation training combined with kinesio taping on walking ability of stroke patients with hemiplegia. *Chinese Journal of Rehabilitation Medicine*.

[27] Lu J. M., Gao T. H., Jia J. (2014). Effect of kinesio taping on lower extremity function after stroke. *Chinese Journal of Rehabilitation Medicine*.

[28] Shao H. X., Ma L., Liu X. M. (2019). Effect of Intramuscular Acombined with Rehabilitation Training on Lower Limb Motor Function, Walking Parameters and Quality of Life in Stroke Patients with Hemiplegia. *Progress in Modern Biomedicine*.

[29] Song W., Wang DQ. (2018). Observation of therapeutic effect of kinesio taping combined with rehabilitation training on stroke patients with foot drop. *Chinese and Foreign Medical Research*.

[30] Sun L. Y., Gao X. M., Sun N. (2015). Clinical observation of different intramuscular patching methods in patients with hemiplegia after stroke. *Medical Information*.

[31] Tan TC., XM Ye., YM Yu. (2016). Effect of kinesio taping on gait in stroke patients with hemiplegia. *Chinese Journal of Rehabilitation Medicine*.

[32] Tang Y., Lin J., Huang XA. (2017). Kinesio taping on bilateral gluteus improves sit-to-stand transfer function in stroke patients. *Zhejiang Medical Journal*.

[33] Wu C., Zhu YL., Liu Q. (2017). Effect of kinesio taping-assisted lower limb training on lower extremity motor function in stroke patients with hemiplegia. *Chinese Journal of Rehabilitation Medicine*.

[34] Xia D. J., Peng T., Wei H. T. (2015). Effect of kinesio taping of lower limbs on walking function in stroke patients with hemiplegia. *Chinese Journal of Physical Medicine and Rehabilitation*.

[35] Xie ZL., Feng SW., Cao QR. (2016). Application of kinesio taping in the prevention of knee extension in patients with hemiplegia after stroke [J]. *Chinese Journal of Rehabilitation Medicine*.

[36] Xu J., SH Hu., Zhou YF. (2016). Clinical observation of kinesio taping with physical therapy on the lower limb function and walking ability in hemiplegics stroke patients. *Chinese Journal of Rehabilitation Medicine*.

[37] Xu L., Li X. P., Gao M. X. (2016). Therapeutic effect of kinesio taping on lower extremity swelling in stroke patients. *Journal of Huaihai Medicine*.

[38] Zhang X., LL Du., Zhao B. (2015). Therapeutic effect of kinesio taping on walking dysfunction in stroke patients with hemiplegia. *Medical Information*.

[39] Chen Z., Chen XQ., Che WJ. (2017). Clinical observation of kinesio taping on the treatment of post-stroke foot inversion. *Journal of Bengbu Medical College*.

[40] Morris D., Jones D., Ryan H., Ryan C. G. (2013). The clinical effects of Kinesio Tex taping: a systematic review. *Physiotherapy Theory and Practice*.

[41] Grampurohit N., Pradhan S., Kartin D. (2015). Efficacy of adhesive taping as an adjunt to physical rehabilitation to influence outcomes post-stroke: a systematic review. *Topics in Stroke Rehabilitation*.

[42] Wang M., Pei Z., Xiong B., Meng X., Chen X., Liao W. (2019). Use of Kinesio taping in lower-extremity rehabilitation of post-stroke patients: A systematic review and meta-analysis. *Complementary Therapies in Clinical Practice*.

[43] Yanhua W., Zhuangmiao L., Xu D. (2018). Effects of kinesio taping at different intervention time on the motor function of lower extremity after stroke: a Meta-analysis. *Chinese Journal of Tissue Engineering Research*.

